# Predictors of the quality of cardiovascular prevention – a multilevel cross-sectional study

**DOI:** 10.3325/cmj.2011.52.718

**Published:** 2011-12

**Authors:** Davorina Petek, Anuška Ferligoj, Rok Platinovšek, Janko Kersnik

**Affiliations:** 1 Department of Family Medicine, Faculty of Medicine, University of Ljubljana, Ljubljana, Slovenia; 2 Faculty of Social Sciences, University of Ljubljana, Ljubljana, Slovenia; 3 Department of Family Medicine, Faculty of Medicine, University of Maribor, Maribor, Slovenia

## Abstract

**Aim:**

To attempt to develop a model of predictors for quality of the process of cardiovascular prevention in patients at high risk of cardiovascular disease (CVD).

**Methods:**

We formed a random sample of patients from a stratified sample of 36 family practice registers of patients at high risk of CVD without diabetes and without established CVD. Data were gathered by chart audit and questionnaires about patient and practice characteristics. We defined the process of care as a dependent variable by principle component analysis and tested the relationship of the process with several independent variables (family physicians’, patients’, and practice characteristics). To study the effects of independent variables (predictors) on the process of care we carried out multilevel regression analysis with the patients constituting the lower level and nested within the family physician/practice (the second level).

**Results:**

Multilevel regression analysis included 645 patients from 36 practices (74.1% from the final sample). Patients’ characteristics that predicted the higher-quality process of CVD prevention were younger age (*t* = -4.94, 95% confidence interval [CI] -0.018 to -0.008) and lower socioeconomic status (*t* = -2.18, 95%CI -0.195 to -0.010). Practice characteristics that predicted the higher-quality process of CVD prevention were smaller practice size (*t* = 2.83, 95% CI 0.063 to 1.166), a good information system for CVD prevention (*t* = 3.15, 95% CI 0.030 to 0.282), and the organization of education on CVD prevention (*t* = 3.19, 95%CI 0.043 to 0.380).

**Conclusion:**

This study shows that the quality of cardiovascular prevention could be measured as a composite outcome and future studies should further develop this approach and test the impact of several practice/patient characteristics on the quality of CVD prevention with the international data.

Prevention of cardiovascular diseases (CVD) is an important task for family physicians. While patients with a low risk of CVD profit mostly from public health activities, high risk patients also need preventive activities provided by their family physicians (1,2). In the countries with a national program of CVD prevention (Slovenia is one of them), these activities and procedures can be highly standardized (3) and, therefore, should be easily measurable. The Slovenian national preventive program for CVD was launched in 2001 and requires preventive check-ups for the defined age groups of patients (women from 45 to 70 years and men from 35 to 65 years). Eighty percent of the target group in every practice needs to go through the program in 5 years, and a register of high risk patients is created in each practice and collected on the national level. The preventive activities consist of two parts: the first part includes a health check-up with determination of risk factors (information on life-style, clinical exam, laboratory tests of lipids and fasting blood glucose) and the second part includes the referral of patients at high risk to preventive workshops, for example for healthy weight reduction, smoking cessation, etc.

Although there is some evidence on several isolated aspects of CVD prevention in Slovenia (4-6), a comprehensive and systematic approach for measuring its quality and actual outcomes is still not available. Therefore, we aimed to develop an integral statistically evaluated presentation of the process of cardiovascular prevention and determine the variables that influence it. Post-hoc analyses were performed on patients at a high risk for coronary diseases using Slovenian data from the international EPA-Cardio study, a cross-sectional study conducted in 9 European countries that had developed quality indicators for cardiovascular prevention on the international level (7) and evaluated the quality of cardiovascular prevention for high-risk patients (8).

## Participants and methods

### Participants

Our aim was to include a sample of 1080 patients at high-risk of developing of CVD in the next decade, ie, 30 patients per practice from 36 stratified randomized practices in Slovenia. The practice was defined as the smallest location or organizational unit of single or several connected practices that could be a part of a larger health care center. The response rate of the practices was 64% (out of 56 invited, 36 agreed to participate). The practices were stratified according to size (small with up to two working family physicians [FP] at the same location and large ones with three or more FPs) and urbanization level (urban practices in settlements with more than 30 000 inhabitants and rural in settlements of 30 000 inhabitants or fewer). The practices from the four stratification groups were chosen by a random number table. Each practice declining to participate had been replaced by a practice under a random number from the same stratification group until 36 practices agreed to participate. Patients were chosen by a set of random numbers from the computerized register of high-risk patients, which every Slovenian practice that follows the National Program of CVD Prevention has to keep. Risk assessment in the program (and for the recruitment of patients in this study) is based on using validated CVD Framingham risk score. High risk for CVD was set at a ≥20% predicted morbidity rate in the next 10 years calculated by the Framingham risk chart. The Framingham risk chart is used by national agreement, although no local validation studies have been performed. We excluded coronary and diabetes patients as their preventive activities and medical care differ from the studied group. Detailed protocol of the international EPA-Cardio study is described elsewhere (8).

### Calculations of the power of the study

A medium level (0.15) of effect size was chosen for unknown R^2^. Based on the estimation of the linear regression analysis sample size with 24 predictor variables and a medium level of the effect size, a sample of 250 participants would be needed in order to achieve 80% power for statistical analysis with an α level of 0.05 (9). Furthermore, the sample needed to be larger than what the above calculation predicts, because of clustering in the data set: patients treated by the same FP are likely to be more similar than patients treated by different FPs. To account for this clustering, we used a multilevel analysis, which achieves lower power than a simple linear regression on disaggregated covariates would.

### Methods

The data were collected using four questionnaires from June 2008 to January 2009. The patients’ data were gathered from the medical records with an audit form. In the audit, we used previously validated quality indicators, which were developed as a first part of the EPA-Cardio project (web-extra material) (web extra material 1) (7). The audit form included questions about the detection and the level of cardiovascular risk factors, as well as their management – especially advice about changes of lifestyle. Patients answered the questionnaires on their demographics and lifestyle, self-rated health, and use of health services. Physicians answered questionnaires on practice characteristics, organization, information technology, and workload. We also performed a semi-structured interview with each physician on his/her preventive work.

For the purpose of analysis, we subdivided the process of CVD prevention into several consecutive steps. The data for the steps 1,3, and 4 were derived from the practice questionnaire, whereas the data for the steps 2 and 5 were derived from the patients’ records. For each step, a compound variable was calculated by adding the number of positive answers of the original dichotomized YES/NO variables, with a higher value of a compound variable implying a better CVD prevention process. This represented a categorization of the items into clinically logical domains of cardiovascular prevention. Frequency distribution was made for each compound variable:

1. Identification of patients for cardiovascular prevention (0-9 points). This compound variable represents a number of “yes” answers to the questions about several methods of screening of patients for CVD prevention: systematic screening for each age group, opportunistic screening for special groups of patients such as overweight patients, patients with diabetes, smokers, hypertensive patients, specific ethnic groups, and low socioeconomic status patients, and screening on patient’s demand.

2. Identification of risk factors (0-6 points). This compound variable represents a number of “yes” answers to the questions about detection of several risk factors for CVD: smoking status, physical activity, calculated body mass index, measurement of blood pressure, identification of cholesterol level, and blood glucose level.

3. CVD risk assessment (0-13 points). This compound variable represents how the practice determines cardiovascular risk: a number of known risk factors that are included in risk assessment; implementation of CVD risk assessment in the practice; the inclusion of the following patient data in CVD risk assessment – age, sex, smoking status, blood pressure, diabetes, family history on CVD, diagnosis on CVD, alcohol consumption, body mass index, lipid status, and fasting/random blood glucose or oral glucose tolerance test; and the use of standardized risk assessment tools in the practice.

4. Process of risk management – physicians (0-4 points). This compound variable represents a number of “yes” answers to the questions about general approach to education on lifestyle: offering written information on healthy lifestyles, advice on Web sites with lifestyle advice, advice on local physical exercise programs, and education on children’s lifestyle.

5. Process of risk management – patients (0-6 points). This compound variable represents a number of “yes” answers to the questions about the physicians’ advice for CVD prevention: data in patients’ medical records in the last 15 months; advice on smoking, physical activity, and nutrition, motivation for quitting smoking, and follow-ups on quitting smoking.

We also determined several independent variables (several characteristics of physicians, patients, and practice) to test their impact on the process quality of CVD prevention. Physicians’ characteristics were age, sex, and professional activity on the projects of CVD prevention.

Practice characteristics were several dichotomous and compound variables: size (small/big) and location (urban/rural), information system for cardiovascular prevention (measured by a compound variable for the completeness of the information in medical records such as problem list, data on regular medication, smoking status, hypertension, diabetes, and/or CVD; system for classification of the disease; standardized risk assessment method), organization of prevention (measured by a compound variable with the active screening of risk patients and a system for recalling patients), availability of the information on CVD prevention for patients, involvement in public health projects on CVD prevention, organization of the education of CVD prevention (compound variable on the practice’s organizing education in which the physicians and nurses take part), and workload (number of patients on the practice list).

Patients’ characteristics were age, sex, education (≤9, 10-13, >13 years), employment status (unemployed/employed, retired, or unable to work), self-evaluation of socioeconomic status (low, middle, high), ethnic origin (Slovenian/other), self-assessment of health (5-point ordinal scale from excellent = 1 point to bad health = 5 points), self-assessed health related to the quality of life (EuroQol instrument – a questionnaire with five domains: mobility, self-care, every-day activities, pain, anxiety/depression – each domain has 3 levels, 1 point for the best status and 3 points for the worst), the length in years of attachment to the same practice, frequency of patients’ visits to the practice per year, the presence of chronic diseases that represent risk factors for CVD (number of “yes” answers), and adherence to prescribed medication therapy (number of “yes” answers in the questionnaire) (10).

Independent compound variables were constructed by a sum of individual variables with a “yes” answer that represented a good approach to cardiovascular prevention. Dichotomous variables were coded yes = 1 and no = 0. Covariates were selected according to the hypotheses. For practice characteristics, we based the selection on a previous study on quality in primary care practices (11). For each variable in a hypothesis an empirical variable was created.

### Statistical analysis

The process of CVD prevention was measured by five compound variables described above. Principal component analysis (PCA) was used to obtain an overall measure of the process of CVD prevention in order to reduce the number of variables and to detect structure in the relationship between variables. By PCA we measured underlying components for five compound variables. The number of components was determined by the highest eigenvalue in the scree plot and also by component loadings, which are correlations between the component and each variable. The PCA analysis was performed using the statistical package SPSS, 17.0 (SPSS Inc., Chicago, IL, USA).

To analyze the relationship of physicians’, patients’, and practice characteristics and the process of CVD prevention, we used a random intercept model. A multilevel model accounts for correlation in the dependent variable for patients treated by the same FP (12). The patients constituted the lower level nested within the FP/practice; the FP/practice presented the same level in our model, because we always had exactly one FP per practice in the sample. Model 0 contained no covariates, model 1 contained patient-level covariates, and model 2 contained covariates measured at the FP/practice level in addition to the patient-level covariates.

We specified the fitted model separately for each of the two levels. The patient-level model closely resembled the simple linear regression model. We gradually entered the explanatory covariates into the model and examined the effect on each variance component. The variance components and model comparisons are given in Table 1. The confidence intervals for the variance components are given in parentheses and were obtained by profiling the likelihood. We performed the estimation in the GNU general Public License software R, using the package lme4a (13,14). The second row of the rightmost part of Table 1 shows the results of the likelihood ratio test of Model 1 against Model 0: Model 1 fits the data considerably better (χ^2^ = 43.4, *P* < 0.001), which is also indicated by other measures of fit, eg, the Akaike information criterion decreasing from 1106.7 to 1089.3.

**Table 1:** Components of the “cardiovascular disease process” variance and model comparisons obtained by entering patient characteristics and practice/physician characteristics covariates in two levels of regression analysis

		Variance							
Model	Description	intercept (95% CI)*	residual (95% CI)*	Akaike information criterion	Log-likelihood	Deviance	χ^2^ test	Degrees of freedom	*P*
0	no covariates	0.679 (0.437-1.129)	0.261 (0.234-0.293)	1106.7	-550.4	1100.7			
1	patient-level covariates	0.678 (0.436-1.129)	0.243 (0.218-0.272)	1089.3	-528.6	1057.3	43.4	13	0.000
2	all covariates	0.349 (0.222-0.584)	0.243 (0.218-0.272)	1088.0	-517.0	1034.0	23.3	11	0.016

*CI – confidence interval.

Adding the covariates measured at the FP/practice level to the model further improves the model fit (χ^2^ = 23.3, *P* = 0.016): the rightmost part the third row of Table 1 shows the results of the likelihood ratio test of Model 2 against Model 1. The effect on the intercept variance was dramatic – it was reduced nearly by half, indicating that the covariates measured at the FP and practice level explain a substantial part of the variability between FPs/practices in the process of CVD prevention. The significance of effects in the model was not evaluated in terms of *P* values, since calculating *P* values for coefficients in a multilevel model can be problematic (14). Rather, we examined the significance on the basis of 95% confidence intervals that were obtained by profiling the likelihood: if the interval does not contain the zero-value, we can be reasonably certain that the direction of the effect is stable.

## Results

### Sampling

**Figure 1:**
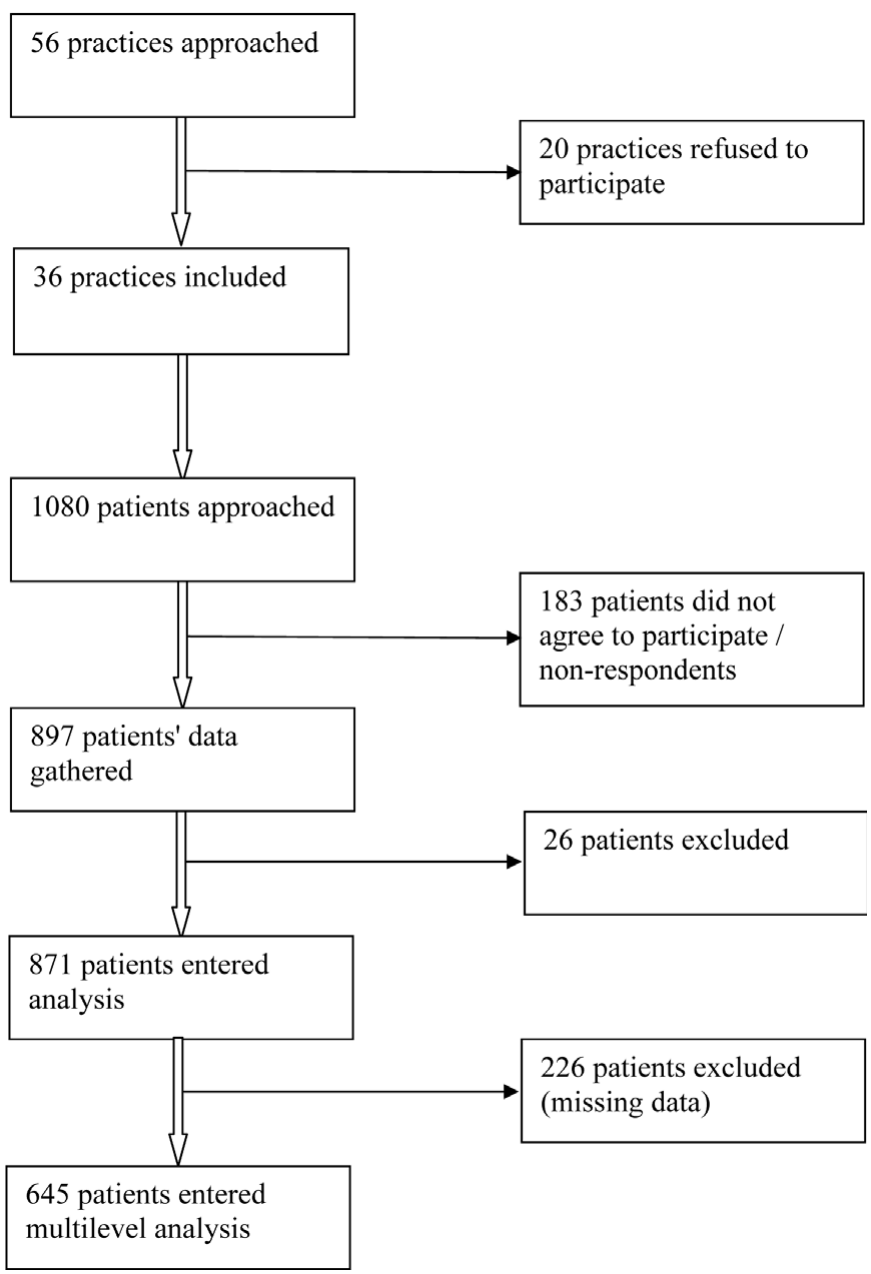
Flowchart of the sampling process of practices and patients enrolled in the study on predictors of quality of cardiovascular prevention in Slovenia

We approached 1080 patients with high CVD risk in 36 practices. The questionnaire was returned by 897 patients, for whom the data were collected from the records. We excluded 26 patients because of unclear coding or not fulfilling of the inclusion criteria. The final sample consisted of 871 patients (80.6% response rate). However, only 645 patients entered multilevel regression analysis while the rest had missing data (74.1% of the final sample). We excluded the units that had a missing value in at least one of the variables in the model. Twenty-two patients were excluded because of unavailable data from the chart audit and the following 72 patients because they did not complete the patient survey. The rest of the 226 exclusions occurred because of sporadic missing in the variables (Figure 1). The original sample and the sample that entered linear multiple regression analysis were similar in structure (Table 2).

**Table 2:** Demographic characteristics of the patients enrolled in the study on predictors of quality of cardiovascular prevention in Slovenia

Demographic characteristics	No. (%) of patients who entered multilevel analysis
Sex (n = 837)	
women	224 (34.7)
men	421 (65.3)
Education (n = 757)	
elementary school or less	231 (35.8)
high school	280 (43.4)
university	134 (20.8)
Employment status (n = 784)	
unemployed	24 (3.7)
other*	621 (96.3)
Socioeconomic status (n = 750)	
low	145 (22.5)
middle	473 (73.3)
high	27 (4.2)
Marital status (n = 779	
married	498 (77.2)
other†	147 (22.8)
Ethnic origin (n = 750)	
Slovenian	603 (93.5)
other	42 (6.5)

*Retired, home-maker, employed, self-employed, unable to work.

†Divorced, widow/er, single.

### Patient characteristics

The mean age of the participants was 62.9 years (95% confidence interval [CI] 62.1 to 63.7), 62.0 years (95% CI 61.1 to 63.0) for men and 64.5 years (95% CI 63.2 to 65.9) for women. Most of the patients finished high school (43.4%). Only 3.7% were unemployed, others were employed or retired or unable to work, and 73.3% estimated their socioeconomic status as average (Table 2). The majority of the patients self-rated their health as good (42.8%), 37.9% as fair, and 9.1% as poor. More than two thirds (68.1%) of patients were visiting the same practice ≥13 years. There were 37.6% of patients who were visiting the practice 4-5 times a year and 22.8% 2-3 times a year.

### Practice characteristics

The 23 included practices were small – two or fewer full time equivalent FPs working at the same location (18 in suburban areas and 5 in urban areas) and 27 were located in the suburban areas (18 small and 9 big). The mean number of the patients on the practice list was 2096 (95% CI 2038.7 to 2153.2). Approximately three quarters (n = 28) of FPs were women with the average age of 47.1 years (95% CI 46.5 to 47.6), while their male colleagues were on the average 53.8 years old (95% CI 53.0 to 54.5). The practices were distributed randomly throughout the country.

### Principal component analysis

We applied the PCA on the compound variables describing the process of CVD prevention (Table 3) in order to identify possible underlying variables, ie, “common denominators” that would enable easier interpretation of the prevention process. The loadings of the original five compound variables on the first principal component were positive and showed relatively high values (above 0.45) (Table 4). We determined the number of components by the highest eigenvalue in the diagram and also by the component loadings, which are correlations between component and each variable.

**Table 3:** The frequency distribution and standard deviation of the five compound variables of the process of cardiovascular disease prevention in 871 high risk patients for cardiovascular disease enrolled in cross-sectional study in Slovenia

Compound variable (number of original variables)	No. of points (mean ± standard deviation)
Identification of patients (9)	4.94 ± 1.70
Identification of risk factors (6)	4.8 ± 1.41
CVD risk assessment (16)	14.6 ± 0.91
Process of risk management – physicians (4)	2.36 ± 0.88
Process of risk management – patients (5)	1.63 ± 1.27

**Table 4:** Principal components loading, eigenvalues, and explained variance in the principal component analysis of the process of the cardiovascular prevention in cross-sectional study of high risk patients for cardiovascular disease in Slovenia

Variables of the process	Components and loadings				
	1	2	3	4	5
Identification of patients	0.59	0.56	-0.09	-0.57	0.09
Identification of risk factors	0.71	-0.52	-0.07	-0.09	-0.47
Risk assessment	0.51	0.54	-0.48	0.47	-0.04
Risk management – physicians	0.48	0.30	-0.79	0.22	-0.03
Risk management – patients	0.68	-0.56	-0.06	0.08	0.46
λ – eigenvalue	1.80	1.27	0.87	0.61	0.44
σ^2^ – percentage of explained variance	36.10	25.44	17.44	12.13	8.90

**Figure 2:**
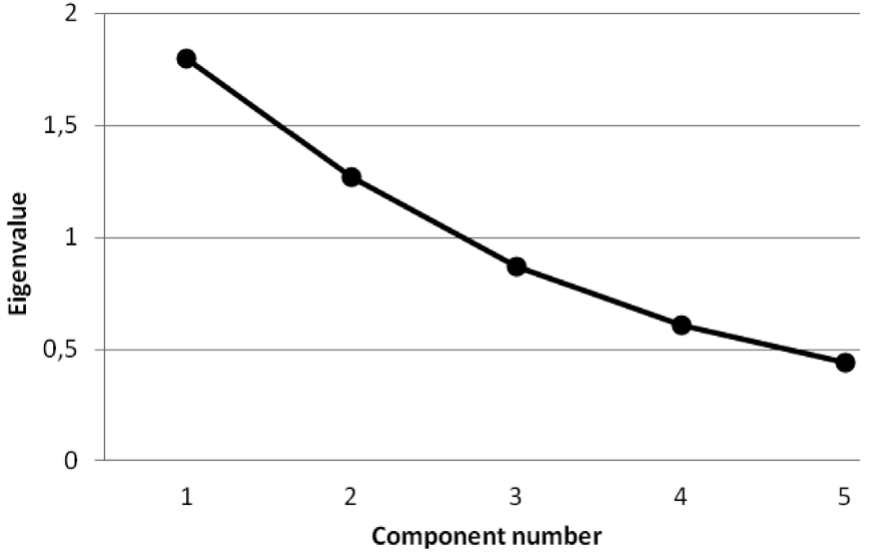
Scree diagram of the principal component analysis of the process of cardiovascular disease prevention in the cross-sectional study of high risk patients for cardiovascular disease in Slovenia

Based on the scree plot (Figure 2), we decided to retain only the first principal component, explaining 36.1% of the total variance. The distribution of the dependent variable “process” was normal, confirmed by the frequency histogram and by the Kolmogorov-Smirnov test (Z = 0.97; *P* = 0.30).

### Multilevel analysis

The impact of the characteristics of physicians, practices, and patients on the process of CVD prevention is presented in Table 5. Patients’ characteristics that were positive predictors of the process of CVD prevention were only younger age (-0.013; 95% CI, -0.018 to -0.008, *t* = -4.94) and lower socioeconomic status (-0.103; 95% CI, -0.195 to -0.010, *t* = -2.18). Practice characteristics that were predictors of the process of CVD prevention were smaller size (0.615; 95% CI, 0.063-1.166, *t* = 2.83), a good information system for CVD prevention (0.156; 95% CI, 0.030-0.282, *t* = 3.15), and the organization of education on CVD prevention (0.212; 95% CI, 0.043-0.380, *t* = 3.19). Two practice characteristics that showed borderline importance were cooperation in programs of CVD prevention and urban practice location.

**Table 5:** Regression coefficients of the multilevel prediction model for the process of cardiovascular prevention with the characteristics of patients, physicians, and practices in cross-sectional study of high risk patients for cardiovascular disease in Slovenia

	Estimate (95% confidence interval)	Standard error	t-value
Intercept **(number of original variables)**	-0.473 (-1.117-0.171)	0.261	-1.82
Patients’ variables (13):			
sex (female)	-0.036 (-0.125-0.053)	0.045	-0.79
age	-0.013 (-0.018 to -0.008)	0.003	-4.94*
education	0.011 (-0.051-0.073)	0.032	0.34
socioeconomic status	-0.103 (-0.195 to -0.010)	0.047	-2.18*
employment (unemployed)	0.095 (-0.122-0.312)	0.110	0.86
marital status (married)	0.057 (-0.040-0.154)	0.049	1.16
ethnic origin (Slovenian)	-0.083 (-0.247-0.080)	0.083	-1.00
self-rating of health	-0.025 (-0.092-0.042)	0.034	-0.73
quality of life	-0.002 (-0.034-0.031)	0.016	-0.09
length of attachment	-0.011 (-0.060-0.037)	0.025	-0.46
frequency of visits	0.017 (-0.016-0.051)	0.017	1.01
chronic diseases	0.019 (-0.037-0.076)	0.029	0.67
regular intake of medication	0.007 (-0.025-0.039)	0.016	0.45
Physicians’ variables (3):			
cooperation in programs of cardiovascular disease (CVD) prevention(yes)	0.401 (-0.076-0.879)	0.188	2.14
physician’s age	0.025 (-0.008-0.058)	0.013	1.90
physician’s sex (female)	-0.032 (-0.650-0.586)	0.243	-0.13
Practices variables (8):			
practice location (urban)	0.425 (-0.111-0.961)	0.211	2.02
practice size (small)	0.615 (0.063-1.166)	0.217	2.83*
information system for CVD	0.156 (0.030-0.282)	0.050	3.15*
organization of prevention	0.072 -(0.164-0.307)	0.093	0.77
access to information	0.080 -(0.415-0.575)	0.195	0.41
cooperation of practice in CVD projects	-0.168 -(0.440-0.105)	0.107	-1.56
organization of education	0.212 (0.043-0.380)	0.066	3.19*
workload in practice	0.000 (0.000-0.000)	0.000	0.24

*Statistical significance (95% confidence intervals not containing the zero-value).

## Discussion

We found that particularly important factors for the CVD prevention were information system of the practice and the ability to capture the complete data in medical records, such as problem list, medication list, or records on risk factors. Another practice characteristic that improved the process of the CVD prevention was organization of continuous education on CVD. Among several patients’ characteristics that were tested, only socioeconomic status and age were important, both pointing to the groups of patients that need more attention.

Our results are in line with the article by Mant (15), who stated that the process measurement was relatively independent of the extrinsic factors such as patient characteristics or their life-style, and is sensitive to differences in the quality of care. We explained an important part of variability of the process of CVD prevention with proposed practice and physicians’ characteristics and only a small part with patient characteristics.

Special attention when conducting preventive activities should be given to patients with poor financial circumstances since a low socioeconomic status has a well known (16,17) and multifactorial (18) influence on higher incidence of CVD, due to a more frequent presence of some risk factors, such as smoking. Also, the most frequently used risk assessment forms (Framingham, Score) are more likely to identify high CVD risk in higher socioeconomic groups (18). Our results showed that FPs are probably aware of these facts, because the socioeconomic status is not part of the system of the CVD prevention and a better process of CVD prevention could be a result of opportunity screening.

One of the most consistent critiques of our national preventive program is that the eligible patients are too old. According to our results, physicians offer a better process of CVD prevention to younger patients who enter the preventive program. Nevertheless, the average age of study patients is 63 years, which makes this conclusion of relative value. The average age of Slovenian participants of the international part of the EPA-Cardio study is similar to other countries (19), but high for primary prevention activities.

Multivariate analyses found that the most numerous and important independent predictors of the process were practice characteristics, such as good information systems, including standardization of risk assessment, systematic organization of medical records, and standard classification systems of the diseases. Other studies have also found that an important predictor is better information technology (20-23).

Another important predictor was organization of education on CVD. Most of the FPs in the country are employed in the public health care centers and their individual education plans are coordinated by the practice management. Better education plays a role in the improvement of the quality of the service. We believe that this is an important finding, which is also in line with the results of other studies: continuous medical education has recently gained high importance in Central and Eastern European countries including Slovenia (24) and a lower availability of continuous medical education options is often perceived by FPs as a potential threat to professional development (25).

Smaller practices showed better quality of CVD prevention. The size of the practices in many European countries has increased in the last decade and larger practices can offer a wider range of activities (26,27). However, smaller practices have to rely on themselves in order to fulfill the obligations to the national preventive program, especially as many of them are private contractors.

No physicians’ characteristic was a predictor of a better process of CVD prevention. One predictor that was close to statistical significance, with high *t* value, was the FPs’ professional activity – working in CVD preventive projects. It is possible that in a larger sample, these characteristics could become an important predictor, as well as location of the practice (urban vs rural).

We presented one possible model for measuring the quality of the process of prevention. It is a broad prediction model, which comprehensively takes into account numerous factors related to the practice, physicians, and patients (28). Using PCA, we also revealed the internal structure of the data. The first component was defined by the identification of the patients and risk factors, risk assessment, and risk management. Therefore, we conclude that the first component measures the overall process of CVD prevention. With the development of the process as a quality-measuring variable represented by defined compound sub-variables, ie, consecutive steps of the process, we could use the majority of the data that are important for the quality in the CVD prevention process.

Another advantage of our approach is that the data came from different sources. The data for the identification of risk factors and risk management came from medical records, but the data on risk identification and methods for risk assessment, and some data on risk management, came from interviews with physicians. Therefore, we were able to assess the quality not only with the data from the medical records, but also by combining physicians’ reports on their performance with the patients’ personal and health characteristics.

A 64% response rate from the selection of practices is enough to claim that the study is representative, although the selection could be biased toward the practices with more interest in prevention activities. Another limitation could be the fact that we used the Framingham risk score for evaluating CVD risk morbidity. It is known that it systematically overestimates the risk of coronary heart disease in populations with lower coronary heart disease mortality (29). Slovenia is still among the European countries with higher coronary heart disease mortality, and no studies evaluate the Framingham risk score specifically for Slovenia. A national agreement was reached to use the Framingham risk score for risk evaluation of CVD in the National preventive program. Therefore, it was feasible to select the patients on the basis of the Framingham risk evaluation.

According to our results, the process of CVD prevention is more or less dependent on the practice organization and its orientation toward preventive services, which are not specifically dependent on patients’ characteristics. Nevertheless, some patients’ groups need special attention, such as those of lower socioeconomic class. On the one hand, this gives us confidence that physicians are taking great pains to provide patients with appropriate care regardless the patients’ individual characteristics, but on the other it seems that full responsibility for the quality of the CVD prevention process rests upon the physicians and practices. However, it is plausible that other patients’ characteristics influence the outcome of the prevention, as we did not study the long-term outcomes of prevention programs (15,30,31) or patient adherence to lifestyle changes (32-34).

Further prospective epidemiological cohort studies should address the question of quality of cardiovascular prevention. On the basis of our results, we cannot conclude how a good process of cardiovascular prevention relates to the outcomes of CVD prevention, measured in healthy life style and regulated risk factors for CVD. We present one possible model for quality evaluation of CVD prevention and this model could be further tested in international studies.
