# Supplementary material for: Predictors of the quality of cardiovascular prevention – a multilevel cross-sectional study
Source: Croat Med J. 2011 Dec;52(6):718–28. doi: 10.3325/cmj.2011.52.718 (PMC3243324; doi:10.3325/cmj.2011.52.718)
Supplement: Supplementary Table 1 [file CroatMedJ_51_s004.pdf]

A list of shortened versions of items from the Epa Cardio instrument

1. Abstraction tool for high risk patients
Patient's gender
Patient's year of birth
Record of smoking status within the past 15 months
Record of motivation to stop smoking in the past 15 months
Record of follow-up motivated patients in next 3 months
Record of offered advice to stop smoking in the last 15 months
Record of physical activity capacity in the last 15 months
Record of advice about physical activity in the last 15 months
Record of BMI in the last 15 months
Record of body weight in the last 15 months
Record of diet advice in the last 15 months
Record of Blood pressure in the last 15 months
Record of individual target BP level
Record of Cholesterol in the last 15 months
Record of Plasma glucose in the last 15 months

2. A questionnaire for the high risk patients
Patient's gender
Patient's year of birth
Years of education
Employment status
Marital status
Presence of clinical conditions:

- High Bloodpressure
- High Cholesterol

Depression
Self-assessment of health (5 point scale from excellent to poor)
Duration of practice involvement
GP-visit frequency per year
Questionnaire on adherence to treatment (Morisky questionnaire)
EuroQol instrument (EQ-5D)
Nationality
Social class

3. Practice questionnaire
Internet access
e-mail access
Anti-virus protection
Practice pc's protected by user-name and password
Computer-supported Patient file system
Computer used for creating medication prescriptions
Procedure for the management of patient information
Procedure for the management of examination results

Procedure for the management of review of examination results
Direct access to medical guidelines
Direct access to medical journals
Direct access to literature data banks
Use of case findings methods to find CVD patients
Staff alowed to contact patients
System for recalling patients with CVD
Specify if yes (CHD, TIA, CVA, PVD)
System for recalling patients with Diabetes
System for recalling patients with Asthma/COPD
System for recalling patients with Hypertension
System for recalling patients at risk for CVD
System for recalling patients at risk of influenza
System for recalling patients for cervix screening
System for recalling patients for breast cancer screening
System for reviewing prescribed medication
Attendance rate for cervix screening
Attendance rate for influenza vaccination
Attendance rate for breast cancer screening
Procedure for prevention of pressure sores
Procedure for prevention of osteoporosis
Procedure for using folium acid
Procedure for smoking cessation
Participation of the practice in life style programmes
Annual report
Quality report
Clinical audit undertaken in last 12 months
Standards set regarding clinical audit
Practice collect data regarding clinical audit
Evaluated results of clinical audit
Improved quality regarding clinical audit
Existence of critical incident register
All nurses attend training on CVD in last 5 years
At least one nurse attend training on CVD in last 15 months
Nurses take part in education about CVD risk factors in schools
GP take part in campaigns on CVD risk prevention
Nurses take part in campaigns on CVD risk prevention
Patient list size
Practice located in town > 30.000 inhabitans
FTE GP's
practice size
doctors-age
doctors-gender

4. Practice interview
The medical record contains:

Details on current medication
Intolerances and contraindications
Smoking status
Diagnosis of hypertension
Diagnosis of diabetes
Diagnosis of CVD
The practice uses Electronic disease classification system
CVD risk assessment implemented for:
Systematic for age groups
Opportunistic for following patients/circumstances:

- diabetes
- clear overweight
- smokers
- cardiovascular risk
- specific ethnic groups
- socioeconomic status
- if ask for risk assessment

Risk assessement includes age
Risk assessement includes gender
Risk assessement includes smoking status
Risk assessement includes blood pressure
Risk assessement includes personal history diabetes
Risk assessement includes diabetes status
Risk assessement includes family history of CVD disease
Risk assessement includes past history of CVD
Risk assessement includes alcohol consumption
Risk assessement includes weight or BMI
Risk assessement includes fasting lipid profile
Risk assessement includes diabetes or impaired glucose regulation
Use of CVD standardized risk assessment tool

- FRAMINGHAM
- SCORE
- PROCAM
- Other CVD assessment tool

CVD risk tool is integrated in medical record system
Record that CVD risk tool has been offered
CVD risk advice is integrated in medical record system
Regular advice to the patients is given about

- Smoking
- Weight
- Physical exercise/activity
- Blood pressure
- Cholesterol
- Lifestyle children

Consultations/advice is offered regularly on

- lifestyle
- written information lifestyle
- websites
- sports club

Team meeting about quality improvement in last 15 months
GPs attend ≥1 training about CVD in last 5 years
One GP per practice attended ≥1training about CVD in last 15 months
Information leaflets about CVD available at the practice for patients
Directory of local prevention activities available locally
Practice participated in Cardiovascular quality improvement projects
Practice participated in project concerning cardiovascular risk management last 2 years
A public health project on CV risk in the practice area in last 2 years
